# A Better Life: Factors that Help and Hinder Entry and Retention in MAT from the Perspective of People in Recovery

**DOI:** 10.13023/jah.0501.06

**Published:** 2023-04-01

**Authors:** Adam D. Baus, Martha Carter, Jennifer Boyd, Erin McMullen, Trey Bennett, Alexandra Persily, Danielle M. Davidov, Christa Lilly

**Affiliations:** West Virginia University School of Public Health; West Virginia Alliance for Creative Health Solutions, Inc., abaus@hsc.wvu.edu; West Virginia Alliance for Creative Health Solutions, Inc.; New River Health Association; West Virginia Alliance for Creative Health Solutions, Inc.; University of Maryland School of Nursing

**Keywords:** Appalachia, medication assisted treatment, opioid use disorder, practice-based research, West Virginia

## Abstract

**Introduction:**

Opioid addiction and opioid-related overdoses and deaths are serious public health problems nationally and in West Virginia, in particular. Medication-assisted treatment (MAT) is an effective yet underutilized treatment for opioid use disorder (OUD).

**Purpose:**

Research examining factors that help individuals succeed in MAT has been conducted from provider and program perspectives, but little research has been conducted from the perspective of those in recovery.

**Methods:**

This study, co-developed with individuals in recovery, took place in West Virginia-based MAT programs using an exploratory sequential mixed methods approach. The survey was open February through August 2021. Data were analyzed late 2021 through mid 2022.

**Results:**

Respondents experienced many barriers to MAT entry and retention, including community bias / stigma, lack of affordable programming, and lack of transportation. Respondents sought MAT primarily for personal reasons, such as being tired of being sick, and tired of having to look for drugs every day. As one respondent shared, *“ I wanted to better my life, to get it under control.”*

**Implications:**

Programs and policies should make it easy for individuals to enter treatment when ready, through affordable and accessible treatment options, reduced barriers to medications, focused outreach and education, individualized care, and reduced stigmatization.

## INTRODUCTION

Opioid-related deaths in the U.S. have increased for the past 25 years and are now at a record high, with 102,429 deaths reported from January 2021 to January 2022.^1^ West Virginia (WV) leads the nation with a drug overdose mortality rate of 81.4 per 100,000 compared to the national average of 21.4 per 100,000.^2^,^3^ While most WV overdoses involve more than one drug, the vast majority (84%) include opioids.^1^ The rates of neonatal abstinence syndrome in WV are also among the highest in the nation.^4^

Medication-assisted treatment (MAT) is an evidence-based, patient-centered approach to treating opioid use disorder (OUD), combining medication with counseling and behavioral therapy.^5^ MAT is effective in decreasing mortality, increasing retention in treatment, decreasing illicit opiate use and criminal activity, supporting employment, and improving birth outcomes among pregnant individuals with OUD.^5^–^7^ However, only 28% of those needing MAT for OUD receive it, and there is a gap of four to seven years on average between onset of OUD and start of treatment.^8^,^9^ MAT is not readily available, especially in rural areas, which are disproportionately affected by the opioid crisis.^10^ Barriers to access include lack of prescribers, long wait times to get into programs, social stigma, cost, transportation, negative prior treatment experiences, peer pressure to continue using, and lack of knowledge of where to receive treatment.^11^–^13^ Retention in MAT is also challenging. Although retention is not defined consistently, it is clear that individuals who remain in treatment longer than a year tend to have better outcomes.^14^–^16^

While significant research has been conducted on MAT from the perspective of healthcare entities, little research has been conducted from the perspective of people in treatment. Understanding the patient perspective is critical in addressing their challenges, understanding their successes, and informing policy.^17^ This study examines factors that facilitate or create barriers to MAT in WV. It was designed in collaboration with people who have lived experience of OUD and have previously participated or are currently enrolled in MAT.

## METHODS

This study used an exploratory sequential mixed-methods approach, wherein qualitative data collection and analysis informed survey development. Survey questions were based on a review of current peer-reviewed literature on facilitators and barriers to MAT in combination with information gained through three semi-structured interviews and follow-up telephone calls with five individuals who have lived experience in MAT, termed Participant Advisors (PAs). Conversations took place in May and June 2020 through secure web-based communications and telephone. An iterative process was used to design and finalize the survey, accounting for readability, reading level, and appropriate language to reflect commonly used and accepted terms. Content validation was also conducted with external reviewers. The final survey included questions examining barriers and facilitators to MAT presented in Likert-type response format with free text fields for added comments.

To reach people in MAT in all areas of WV, licensed WV MAT programs were recruited for participation using data from the WV Department of Health and Human Resources, Office of Health Facility Licensure and Certification. The study was made available to MAT locations throughout WV in an attempt to represent urban, small town, and rural practices, include programs offering different MAT modalities, reflect racial and ethnic diversity, and include special populations such as those recently incarcerated or pregnant. Programs agreeing to participate received the survey and study materials in paper-based and electronic formats. A cover letter offered information about the study and implied consent to participate. MAT program sites were offered $100 for distributing surveys, and respondents were eligible for a $200 gift card prize drawing. Prize drawing data were collected securely and not connected to the survey responses. The survey was open from February through August 2021. All survey responses are patient and site anonymous to ensure patient confidentiality.

Quantitative data were analyzed via descriptive and inferential statistics using SAS Analytics Software. Descriptive analyses included valid percentages and frequencies of categorical data and means and standard deviations of continuous data. Four item sets were ranked according to items most endorsed and examined via Kendall’s tau statistic for differences by gender and time in MAT. Qualitative data were analyzed via content analysis using inductive, open coding and synthesis. Categories were operationally defined, paired with accompanying relevant quotes, and reviewed, with discrepancies resolved through discussion and consensus. Data were analyzed late 2021 through mid 2022. This study received West Virginia University Institutional Review Board Approval (Protocol #2001837052).

## RESULTS

Twenty-one (21) WV MAT programs were recruited, with 1,700 surveys distributed, resulting in 225 responses (13.2% response rate), which included over 500 free-text comments. The majority of responses were paper based (n=196, 87.1%). All quantitative results are presented in terms of valid percent. Qualitative findings from semi-structured interviews and open-ended survey questions are intermixed for added context.

### Demographics and Other Social Factors

The majority of respondents were White (n=205, 95.8%), non-Hispanic (n=177, 96.7%), aged 35–54 years (n=108, 50.9%), and evenly distributed by gender identity (female n=109, 51.2%; male n=102, 47.9%). Medicaid (n=136, 66.7%) and Medicare (n=40, 19.6%) represented the majority of coverage for MAT services, while a minority of respondents paid full cost out of pocket or via sliding scale. Demographics and other social factors are presented in Table 1.

### Drug Use and Treatment History

Respondents reported long-term drug use and participation in a variety of treatment options. Roughly half (n=107, 52.2%) reported drug use had been a problem for 11 years or more. Almost all (n=207, 95.0%) were currently enrolled in MAT. Nearly half (n=91, 44.9%) had been in MAT for longer than two years. Results indicate that 44 (19.6%) respondents decided to go into treatment after overdosing and 15 (6.7%) received treatment referral by a harm reduction program or quick response team.

Respondents engaged in multiple types of OUD treatment over time and on average had tried three treatment options. Suboxone (buprenorphine/naloxone) was used by nearly all respondents (n=207, 95.0%), followed by buprenorphine alone (n=41, 18.2%), methadone (n=32, 14.2%), and naltrexone (n=27, 12.0%).

Many (n=95, 42.2%) reported difficulty getting into any type of treatment. Most (n=110, 51.4%) indicated that it is “very important” to receive inpatient (hospital) treatment for OUD, while 27.1% (n=58) responded that inpatient treatment is at least “slightly important.” Fifty respondents (26.6%) reported difficulty getting a prescription for MAT, including delays in Medicaid/insurance approval (n=34, 39.1%), cost of medication (n=31, 35.6%), medication availability at pharmacies (n=30, 34.5%), and lack of transportation (n=20, 23.0%). One respondent wrote, “*I did not have insurance and I could not afford to pay for treatment. Once I got insurance and could get into treatment, I had a hard time finding a place to go to nearby in West Virginia.”* Some reported that being pregnant prevented them from getting into MAT (n=6, 6.2%). Among respondents who had been in jail or prison related to drug use (n=70, 33.8%), only 18 (13.5%) received MAT in a correctional setting and only 23 (19.8%) received help getting into a treatment program upon release.

Forty-four (44) respondents (22.2%) reported being kicked-out or discharged from MAT. Nearly half indicated that they were discharged because they “used” and that was against program rules (n=21, 56.8%). Others were discharged for missing appointments (n=10, 27.0%) or because they could not follow other program rules (n=5, 13.5%). One respondent added, “*At first it was difficult to get time off with work to meet requirements at the program. It was hard to get a set day off every week to see the doctor plus keep up with required therapy appointments.”*

Nearly two-thirds (n=125, 62.2%) reported purchasing non-prescribed Suboxone, most commonly to avoid withdrawal (n=85, 61.6%) and inability to get into a treatment program that prescribed Suboxone (n=60, 43.5%). One respondent wrote, “*I had a fear that even with the treatment, I would still crave drugs and get sick. I tried it on the street before committing to the program to make sure I could do it. I was thrilled that I wasn’t sick or craving anything.”* Additional drug use and treatment history findings are presented in Table 2.

### MAT Entry and Retention

Four questions were posed regarding entry into and retention in MAT. Results from these questions were ranked and examined for variance based on time in treatment and gender. No differences were detected that were both statistically significant and significant from a policy or programmatic perspective. The five highest ranked responses are provided, with accompanying free-text highlights.

- “Why did you decide to go into a MAT program?”
  ○ Top five responses: “I was tired of being sick; I was tired of having to look for drugs every day; I found a program that has appointment times that work for me; I got into a program I could afford; I got into a program the day I was ready.”
    ■ *“I was tired of hunting drugs, never having money, having to hide from loved ones who weren’t users, and just tired of the situation in general.”*
    ■ *“I found out I was pregnant and wanted to do the right thing. I was sick and tired of living that way and knew there was a better way to live.”*
- “What kept you from going into a MAT program?”
  ○ Top five responses: “I wasn’t ready; Community bias or stigma against addiction treatment; Community bias or stigma against MAT; My 12-step program judged me for using medications to treat addiction; I didn’t have a way to pay for treatment.”
    ■ *“There were several factors that stopped me from entering treatment. One was I didn’t have insurance, the second reason I wasn’t ready to quit.”*
    ■ *“I was scared and didn’t want anyone to find out. Whether it’s right or wrong people do judge, and I didn’t want that on top of everything else. I can’t say this enough, but I went on my time, when I knew I had enough. It wasn’t mandated, I just knew I needed help.”*
- “What helps you stay in a MAT program?”
  ○ Top five responses: “The medication is working for me; Individual counseling helps me; I feel normal for the first time in a long time; I make good connections with people who run the program; I have good support from family.”
    ■ *“This program is the reason I can lead a normal life. I have been able to hold down a job. The program has allowed me to have relationships with family and friends…simply put saved my life!”*
- “Why did you – or would you – leave a MAT program?”
  ○ Top five responses: “I didn’t have a way to pay for treatment; I didn’t have good transportation; I still had cravings; Judgmental attitudes of people running the MAT program; I couldn’t get away from old friends and habits.”
    ■ *“The only way I would leave is if I lose Medicaid because I feel I am on the treatment for life. Don’t feel safe without it.”*
    ■ *“I haven’t left a MAT program, but not having good transportation makes it difficult to get to appointments and I’ve been close to getting kicked out.”*
    ■ *“At some point would like to consider reducing dose and stopping treatment but it is too hard to get back in treatment if you leave. That affects my decision.”*

**Figure 1:**
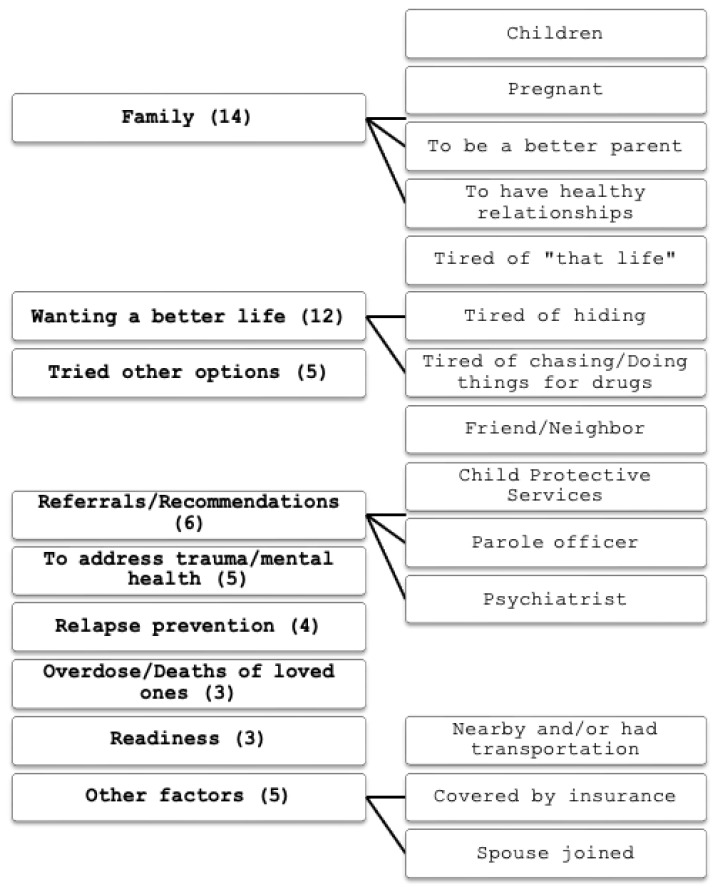
Additional comments for deciding to enter MAT by category NOTE: Categories of additional free text comments on factors that supported their decision to enter MAT.

**Figure 2:**
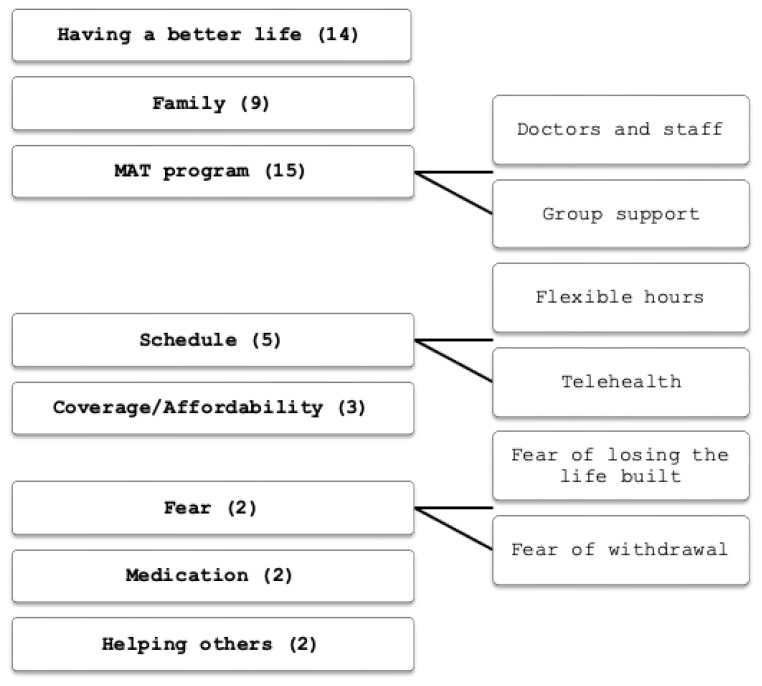
Additional comments for staying in MAT by category NOTE: Categories of additional free text comments on factors that supported their decision to stay in MAT.

MAT entry and retention findings are presented in Table 3. Supplemental qualitative findings derived from the free text survey questions are presented in Fig. 1 and Fig. 2.

## DISCUSSION

This research aims to better understand factors that help and hinder individuals seeking MAT in WV from the perspectives of people in recovery. The main factors that help people get into treatment are personal. Yet the findings point to significant policy and programmatic elements that facilitate participation in MAT when the individual is ready to seek treatment. There is an ongoing need for public education to address commonly held misunderstandings about addiction and reduce stigma regarding treatment. Treatment must be delivered respectfully, supporting the agency of those in recovery, and address social, environmental, and other factors supportive of recovery. As one PA commented about choosing a treatment program, “*You’ve got to be comfortable. You’re putting your life in these people’s hands.”*

### Insurance Coverage

Aligned with national trends, Medicaid is the largest payer of MAT services in the study.^18^ A major benefit of Medicaid is that medications, including MAT, are available to beneficiaries with minimal or no co-pays due to federal requirements that cap out-of-pocket costs. Commercial insurers are not subject to similar caps on co-pays. Even small copays are associated with decreased use of medically necessary care.^19^ Furthermore, many insurers employ utilization management policies that can delay access to MAT.^19^ Among those who reported difficulty in accessing MAT prescriptions, the majority noted that this was due to delays in approval from Medicaid or other insurers. Payers should examine their policies to reduce barriers to MAT, including eliminating prior authorization and copays for MAT. States, insurers, and programs should bolster efforts to inform individuals of insurance eligibility, including Medicaid, and coverage for SUD treatment and transportation. Outreach should target special populations including pregnant individuals, who may face additional stigma and barriers to care when seeking MAT.^4^,^20^

### Access to Treatment

Although recent federal policy has supported expanded access to MAT,^19^, ^21^–^22^ many parts of the U.S. continue to have a limited supply of MAT providers and therefore insufficient opportunities for treatment.^23^ Additionally, despite the high importance respondents placed on inpatient drug treatment, federal Medicaid policy generally places limitations on payment for inpatient SUD treatment in facilities larger than 16 beds.^24^ The substantial number of respondents who reported difficulty getting into any type of treatment highlights the significance of these structural barriers to care.

A major barrier to MAT prescribing was recently addressed by the Consolidated Appropriations Act of 2023.^25^ This legislation removed the requirement for practitioners who hold a Drug Enforcement Agency license to obtain a waiver, commonly called the Drug Addiction Treatment Act of 2000 (DATA 2000) or X-Waiver, to prescribe buprenorphine. We urge practitioners to incorporate MAT into their practices. Improving health professions medical education about OUD can encourage clinicians to integrate effective OUD treatment into practice and reduce stigma associated with treatment.^26^,^27^ Additionally, programs like WV’s hub-and-spoke model, known as the Comprehensive Opioid Addiction Treatment (COAT) program, can play a pivotal role in supporting programs and prescribers offering evidence-based MAT.^28^

MAT access can be improved by offering same-day appointments and tailoring appointment times to meet participant needs. Offering telehealth visits for prescribing and counseling should be included whenever possible. Improved referral systems and connections with community partners are needed to ensure that people who use drugs receive frequent and repeated offers to enter treatment.

### Access Among the Incarcerated/Formerly Incarcerated

Most respondents who had been in jail or prison related to drug use did not receive MAT in a correctional setting and did not receive help in getting into a drug treatment program upon release. As of 2022, this is in opposition to the Americans with Disabilities Act. Moreover, not providing MAT increases risk of overdose among individuals leaving jail or prison.^29^–^31^ Often referred to as “the inmate exclusion,” federal law largely prohibits Medicaid reimbursement for services provided to individuals in correctional settings. This exclusion is a barrier to comprehensive SUD services for justice-involved individuals, including those on parole and probation.^31^ Treatment for OUD should be initiated or continued for these individuals and provisions should be made to ensure continuity of care upon reentry to the community.

### Diversion as Harm Reduction

Most respondents reported that they had purchased Suboxone not prescribed to them. However, only a few bought non-prescribed Suboxone because they wanted to get high. While purchase of non-prescribed Suboxone is a concern for prescribers and programs, it is also a form of harm reduction allowing individuals to avoid withdrawal and overdose. As one PA commented, *“I am so tired of burying my friends. And I have yet to bury somebody who overdosed on Suboxone.”* Improving access to MAT can reduce the use of non-prescribed Suboxone.^32^

### Individualizing Treatment

Respondents indicated that the most important factor helping them stay in MAT is that the medication is working for them. PAs and survey respondents stressed the importance of individualizing treatment and having multiple options available. Prescribers and patients should engage in shared decision making to determine the most effective medication, using up to date, evidence-based dosage/treatment guidelines. Payers should support prescriber discretion and remove barriers to dosing adjustments. Efforts to increase access to individual counseling should be supported, as it was the second most important factor supporting retention.^18^

### Addressing Relapse

Unfortunately, these findings demonstrate that individuals with OUD who relapse are often discharged from MAT programs and denied access to medications. Discharging MAT participants for using illicit drugs clearly diminishes the potential benefit of MAT to people with OUD. MAT programs should closely examine policies related to patient discharge and ensure that options such as referral to a higher level of care or to ancillary services, such as transportation and supportive housing, are offered before discharge.^15^

### Enhancing Peer Support Groups

Judgment from 12-step programs for using MAT was a major reason respondents did not enter or considered leaving MAT. While attendance at 12-step groups is valued by MAT participants and Narcotics Anonymous recognizes that individuals may choose medication during recovery,^33^,^34^ studies also show that 12-step programs often stigmatize MAT.^35^ This suggests that required attendance in 12-step programs should be evaluated from the perspective of people in recovery. Peer support programs should evaluate their positions on MAT based on current evidence and avoid a stigmatizing atmosphere.

### Reducing Stigma

Effective, ongoing public education efforts are vital to combat stigma among the general public and in professional communities, including healthcare professionals. Recognizing OUD as a chronic disorder similar to other chronic disorders such as diabetes, using non-stigmatizing language, understanding reasons for use of non-prescribed Suboxone, and recognizing the value of individualized treatment are ways to reduce stigma and enhance treatment.

### Limitations

The results of this research may not reflect the experiences of those with OUD who have never received MAT, and the experiences of people using methadone may be under-represented. These results best reflect the experiences of patients from Community Health Centers, given the proportion of these organizations among those agreeing to participate. The findings may not apply to other states or regions outside central Appalachia. While this study endeavored to reach diverse populations, results may not reflect the experiences of people who identify as Hispanic, LGBTQ+, and people of color. Lastly, the response rate may affect generalizability. However, the responses received reflect MAT program participant experiences over many years and in a wide range of programs.

## IMPLICATIONS

While the decision to enter treatment for OUD is personal, changes can be made in policy and programs to facilitate entry into treatment without delay when the individual is ready. Increased access to prescribers, no- or very low-cost treatment programs, reduced barriers to medications, assistance with transportation, focused outreach and education, individualized care, and reduced stigmatization can increase entry and retention in MAT programs and reduce opioid overdoses. The voices of those in recovery can highlight the challenges and clarify the solutions to strengthening a system of care for OUD.

**SUMMARY BOX**

**What is already known about this topic?**

Prior research demonstrates the effectiveness of medication assisted treatment (MAT) for opioid use disorder (OUD), and highlights known barriers to individuals entering and staying in treatment. However, more research is available from the perspective of programs and providers than from the perspective of individuals in treatment.

**What is added by this report?**

This study gives a voice to individuals in recovery by learning directly from them factors that facilitate and hinder MAT for OUD, and yields insights into changes that can be made from policy and program perspectives to support recovery. Strengthening a system of care for OUD is possible through acknowledging the importance of personal readiness to enter treatment, removing structural barriers to care, and improving linkage to available resources such as payment support to enter treatment.

**What are the implications for future research?**

Future research should continue to learn from individuals in recovery and monitor progress in strengthening a system of care for OUD and reducing stigma associated with this chronic disorder.

## Figures and Tables

**Table 1:** Demographics and social factors

Response	n	Valid %
**Age category**		
18–34 years	83	39.2
35–54 years	108	50.9
55+ years	21	9.9
**Gender identity**		
Female	109	51.2
Male	102	47.9
Transgender	2	0.9
**Race**		
American Indian or Alaska Native	1	0.5
Black or African American	5	2.3
Multiracial	3	1.4
White	205	95.8
**Ethnicity**		
Hispanic, Latino/a, or Spanish	6	3.3
Non-Hispanic, Latino/a, or Spanish	177	96.7
**Payment source (check all)**		
Medicaid	136	66.7
Medicare	40	19.6
Private insurance or insurance through employer	32	15.7
Full cost out of pocket	25	12.2
Sliding fee scale	9	4.4
Medicaid plus Medicare	8	3.9
**Working right now**		
No	118	58.1
Yes full-time	61	30.1
Yes part-time	24	11.8
**If no, reason(s) for not working (check all)**		
Disabled or unable to work	41	34.7
Being in drug treatment takes too much time	22	18.6
Afraid my employer won’t be flexible enough to allow me to get to my appointments	16	13.6
Afraid my employer will be judgmental of me being in drug treatment	10	8.5
Afraid I’ll start using again	7	5.9
**Used the non-emergency medical transportation system paid for by Medicaid**		
No	150	74.3
Yes	52	25.7
**Health system representation among participating organizations**		
Community health centers/federally qualified health centers	11	52.4
Hospitals/hospital affiliates	3	14.3
Comprehensive behavioral health centers	2	9.5
Free clinics	2	9.5
Private, faith-based organization	1	4.8
Private medical group practice	1	4.8
Private psychiatric practice	1	4.8

**Table 2:** Drug use and treatment history

Response	n	Valid %
**Duration of drug use as a problem**		
Up to 2 years	10	4.9
2 to 10 years	88	42.9
11+ years	107	52.2
**Currently in a MAT Program**		
Yes	207	95.0
No	11	5.0
**Duration in MAT over time**		
<2 weeks	11	5.4
2 weeks to 3 months	23	11.3
>3 months to 12 months	48	23.6
>1 year to 2 years	30	14.8
>2 years to 5 years	53	26.1
>5 years to 9 years	20	9.9
>9 years to 20 years	17	8.4
>20 years	1	0.5
**Medications ever used for MAT (check all)**		
Buprenorphine/Naloxone	207	95.0
Buprenorphine alone	41	18.2
Methadone	32	14.2
Naltrexone	27	12.0
**Types of treatment utilized (check all)**		
Outpatient MAT program	176	78.2
Detox facility	111	49.3
Residential treatment program	93	41.3
Inpatient (hospital) treatment facility	91	40.4
Intensive outpatient program	85	37.8
Outpatient program without MAT	71	31.6
Sober living setting	63	28.0
**Self-Detox (check all)**		
Detoxed on your own and did not go into a drug treatment program	132	58.7
Detoxed on your own before entering a drug treatment program	127	56.4
**Trouble getting into any treatment programs**		
No	130	57.8
Yes	95	42.2
**Problems getting prescription for drug treatment**		
No	138	73.4
Yes	50	26.6
**If yes, reasons for problems in getting prescriptions for drug treatment (check all)**		
Delay in getting Medicaid or insurance approval	34	39.1
Didn’t have the money to pay for my medication	31	35.6
Pharmacy didn’t have the medication, but I was able to get it from another pharmacy	30	34.5
Didn’t have transportation to get to my appointment to get refill prescription	20	23.0
Didn’t have transportation to get to the pharmacy to pick-up medication	18	20.7
Pharmacy didn’t have the medication, and I couldn’t go to a different pharmacy because of program or insurance	17	19.5
Problem getting refill order on time from prescribing provider	15	17.2
Problem getting an appointment to get a refill	4	4.6
**Ever kicked out of or discharged from MAT**		
No	154	77.8
Yes	44	22.2
**If Yes, reasons for being kicked out or discharged from MAT**		
I used, and that was against program rules	21	56.8
I missed too many appointments	10	27.0
I could not follow other program rules	5	13.5
I needed more intensive treatment (higher level of care)	1	2.7
**Ever bought non-prescribed Suboxone**		
Yes	125	62.2
No	76	37.8
**If yes, reasons for buying non-prescribed Suboxone (check all)**		
I wanted to avoid withdrawal	85	61.6
I couldn’t get into a program that prescribed Suboxone	60	43.5
I couldn’t get the drug I wanted to use	28	20.3
I ran out of Suboxone that was prescribed for me	19	13.8
I wanted to get high	10	7.2
**Been in jail or prison for any offense related to drugs**		
No	137	66.2
Yes	70	33.8
**If yes, while in jail or prison were you in a drug treatment program**		
Yes	18	13.5
**If yes, as you were getting out of jail or prison was there help in getting into a drug treatment program?**		
Yes	23	19.8

**Table 3:** Rank scores for facilitators and barriers to MAT

Why did you decide to go into a MAT program?				
Response	Rank	n	Mean	SD
I was tired of being sick	1	213	4.4	1.0
I was tired of having to look for drugs every day	2	211	4.3	1.1
I found a program that has appointment times that work for me	3	205	4.3	1.0
I got into a program I could afford	4	205	4.2	1.0
I got into a program the day I was ready	5	216	4.1	1.1
I had transportation to get to appointments	6	209	4.1	1.2
I got a prescription the day I was ready	7	213	4.0	1.2
I knew someone who had a good experience	8	176	3.8	1.3
I was referred by my medical provider	9	170	2.9	1.5
My family told me I had to get treatment	10	178	2.7	1.5
My friends told me I had to get treatment	11	178	2.4	1.4
I overdosed and decided to get treatment	12	161	2.3	1.5
I was referred by another program such as Harm Reduction or a Quick Response team	13	152	1.8	1.1
I was given a choice between jail or prison and a MAT program	14	134	1.6	1.1
**What kept you from going into a MAT program?**				
Response	Rank	n	Mean	SD
I wasn’t ready	1	176	3.2	1.4
Community bias or stigma against addiction treatment	2	172	3.1	1.4
Community bias or stigma against MAT	3	170	3.0	1.4
My 12-step program judged me for using medications to treat addiction	4	137	2.9	1.5
I didn’t have a way to pay for treatment	5	163	2.8	1.5
The program(s) I tried to get into were not taking new patients	6	152	2.7	1.3
I didn’t have good transportation	7	161	2.6	1.4
Wait time for the program was too long	8	165	2.5	1.3
I couldn’t follow program rules because of my work schedule	9	143	2.5	1.2
I thought the program rules would be too hard to follow	10	167	2.4	1.2
I didn’t have childcare	11	137	2.4	1.3
I didn’t have support from friends	12	171	2.3	1.3
Program hours didn’t work for me	13	168	2.3	1.1
I didn’t have support from family	14	177	2.2	1.3
**What helps you stay in a MAT program?**				
Response	Rank	n	Mean	SD
The medication is working for me	1	206	4.5	0.9
Individual counseling helps me	2	212	4.3	1.0
I feel normal for the first time in a long time	3	209	4.3	1.0
I make good connections with people who run the program	4	211	4.3	0.9
I have good support from family	5	215	4.2	1.0
I am scared of going through withdrawal	6	202	4.2	1.2
I have good support from friends	7	210	4.1	1.0
I have transportation to get to appointments	8	210	4.1	1.0
I get help with other medical issues	9	199	4.1	1.0
I make good connections with other people in the program	10	204	4.0	1.0
I get help with my mental health	11	192	4.0	1.1
Group counseling helps me	12	200	4.0	1.2
I have help from a Peer Support or Recovery Coach	13	190	3.9	1.2
I can afford the cost	14	199	3.9	1.1
I have good support outside the MAT program, such as NA or AA	15	181	3.8	1.2
I am scared of overdosing	16	190	3.5	1.5
I get help with housing, food, or clothing	17	156	3.2	1.4
I get help with transportation	18	158	3.0	1.4
I get help with dental care	19	167	2.9	1.4
I get help with childcare	20	119	2.6	1.3
**Why did you – or would you – leave a MAT program?**				
Response	Rank	n	Mean	SD
I didn’t have a way to pay for treatment	1	183	3.0	1.5
I didn’t have good transportation	2	172	2.8	1.4
I still had cravings	3	194	2.6	1.4
Judgmental attitudes of people running MAT program	4	191	2.6	1.4
I couldn’t get away from old friends and habits	5	188	2.5	1.4
My 12-step program judged me for using medications to treat addiction	6	153	2.5	1.4
Program hours didn’t work for me	7	192	2.4	1.3
I wasn’t ready to stop using	8	191	2.4	1.5
Community bias or stigma against MAT	9	185	2.3	1.4
Community bias or stigma against addiction treatment	10	189	2.3	1.4
I didn’t have childcare	11	132	2.2	1.3
The program rules were too hard to follow	12	196	2.2	1.2
I didn’t have support from family	13	190	2.2	1.2
I didn’t have support from friends	14	186	2.1	1.2
